# Comparison of TPLO tibial tuberosity fractures with and without an in situ rotational pin

**DOI:** 10.1186/s13104-018-3474-7

**Published:** 2018-06-08

**Authors:** Matthew J. Morgan, Jean K. Frazho

**Affiliations:** Department of Surgery Affiliated Veterinary Specialists, 9905 S US Hwy 17-92, Maitland, FL 32751 USA

**Keywords:** Tibial tuberosity fracture, Tibial plateau leveling osteotomy, Reduction pin

## Abstract

**Objective:**

Cranial cruciate ligament (CCL) rupture is a common cause of pelvic limb lameness in dogs. The tibial plateau leveling osteotomy (TPLO) is a well-described surgical procedure that treats CCL ruptures. The objective of this study was to compare the risk of tibial tuberosity fractures from TPLO procedures using a TPLO reduction pin in situ versus patients with a TPLO reduction pin removed at the time of surgery. Our hypothesis is that patients with a TPLO reduction pin left in situ will have a decreased incidence of tibial tuberosity fractures.

**Results:**

A total of 400 dogs that fitted the criteria of 200 consecutive TPLO surgeries performed with each group were included in the study. The Student’s t-test revealed a statistically significant difference in fractures observed in group 1 (in situ pin) and group 2 (no pin). In univariate logistic regression analysis, only the covariate for the presence of the reduction pin was associated with a statistically significant reduction in the likelihood of tibial tuberosity fracture. In the multivariate model, the presence of the reduction pin was associated with an approximate 92% reduction in the likelihood of tibial tuberosity fracture.

**Electronic supplementary material:**

The online version of this article (10.1186/s13104-018-3474-7) contains supplementary material, which is available to authorized users.

## Introduction

Cranial cruciate ligament (CCL) rupture is one of the most common causes of pelvic limb lameness in dogs [1]. The exact etiology of CCL ruptures is poorly understood, but degenerative, biological, mechanical, heritable, and immune-mediated factors have all been considered to be causes of CCL disease [2–4]. The tibial plateau leveling osteotomy (TPLO) is a well-described and popular surgical procedure that treats CCL ruptures by neutralizing the cranial tibial thrust through a radial osteotomy [5]. Complications reported from the TPLO procedure range from 10–34% and include infection, dehiscence, plate and screw breakage, patellar tendonitis, avulsion fracture of the tibia, fracture of the tibia or fibula, meniscal tear, and delayed union [7–9].

Avulsion fractures of the tibial tuberosity have been reported to occur in 1–9% of patients following a TPLO and may contribute to an increased morbidity and the need for revision surgery [10]. It has been hypothesized that the cranial positioning of the osteotomy, large tibial plateau angle (TPA) corrections, inaccurate reduction of the osteotomy gap, oversized saw blade, relative placement of the antirotational TPLO reduction pin, and simultaneous bilateral TPLO surgeries are all risk factors for tibial tuberosity fractures [11–13].

Several surgeons from this institution have elected at times to leave the temporary TPLO reduction pin in situ following completion of the TPLO plate application. Although the placement of the temporary anti-rotational TPLO reduction pin in relation to tibial tuberosity fractures has been studied, no study to date has evaluated if a TPLO reduction pin left in situ will have a protective effect on the tibial tuberosity fractures during the initial 8 weeks of healing [12].

The objective of this study was to compare the relative risk of tibial tuberosity fractures from TPLO procedures using a TPLO reduction pin in situ versus patients with a TPLO reduction pin removed at the time of surgery. Our hypothesis is that patients with a TPLO reduction pin left in situ will have a decreased incidence of tibial tuberosity fractures.

## Main text

### Materials and methods

Medical records for dogs treated with TPLO surgeries from January 2011 to May 2017 that had a TPLO surgery were reviewed. Dogs were included if a TPLO was performed with at least three sets of properly positioned orthogonal radiographs from immediately pre-operatively, post-operatively and at least 8 weeks post-operatively. Dogs were excluded if concurrent procedures were performed on the tibia (e.g. tibial transposition, tibial translocation or tibial cranial closing wedge). No bilateral single session TPLO surgeries were performed during this time period. All second side TPLO surgeries were performed at least 8 weeks post-operatively following radiographic confirmation of a healed osteotomy. All TPLO surgeries included were performed by ACVS board-certified surgeons who had more than 4 years of experience in performing the TPLO.

Records were selected chronologically and sequentially for patients with an in situ reduction pin (group 1) and without an in situ reduction pin (group 2) until 200 stifles were indentified in each group. For each patient included in the study, we recorded the weight, age at the time of surgery, implants used and complications up to the 8 week recheck examination.

**Fig. 1:**
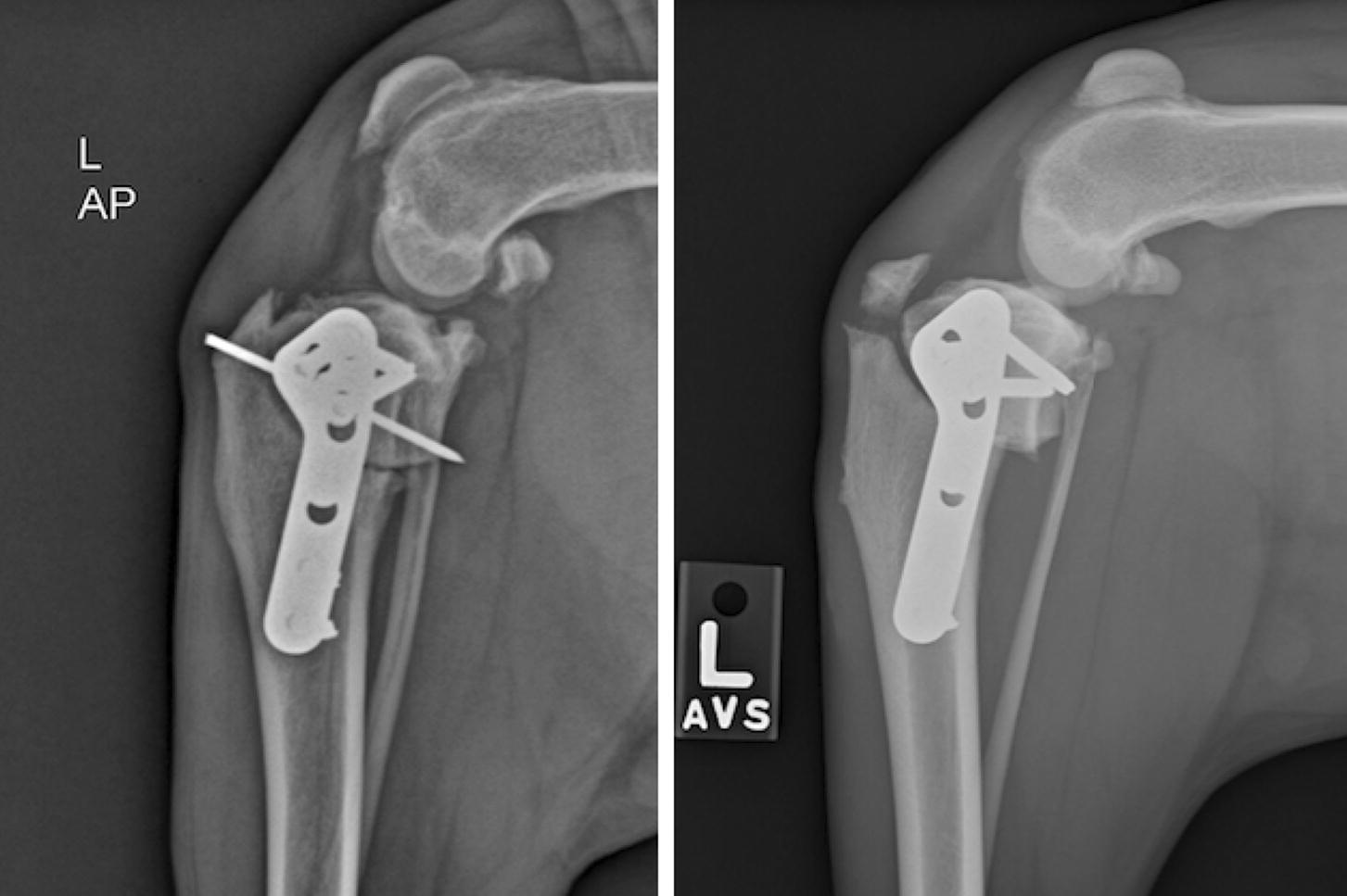
Left image is a TPLO with reduction pin in situ. Right image is a TPLO with tibial tuberosity fracture 4 weeks post-op

TPLO surgeries were all performed with a locking TPLO plate (Veterinary Orthopedic Implants, St. Augustine FL) in the following four sizes: 3.5 mm broad, 3.5 mm regular, 3.5 mm mini, or 2.7 mm. In the proximal segment locking cortical bone screws were used for all available locking holes. In the distal segment non-locking cortical bone screws were used for every surgery. A 5/64″ positive profile end threaded stainless steel pin (IMEX Veterinary Inc, Longview TX) was used as the TPLO reduction pin as seen in Fig. 1.

The craniocaudal and mediolateral radiographs were reviewed at all the three time periods (pre-op, post-op, 8 weeks post-op). Pre-operative radiographs were evaluated for TPA and maximal distance from the tibial tuberosity to the caudal tibial plateau. Immediate postoperative radiographs were evaluated for TPA and the narrowest width of the tibial crest (absolute tibial tuberosity width), defined as the width at the narrowest point of the tibial crest cranial to the osteotomy and distal to the insertion of the patellar ligament on the tibial tuberosity. The relative tibial tuberosity width was obtained by dividing the absolute tibial tuberosity width by the craniocaudal width of the tibia as previously defined by Bergh et al. [11]. This was calculated to be able to compare the relative tibial tuberosity size for dogs of various weights. Measurements were performed, verified and calculated by two authors (MM & JF). All radiographs evaluated were calibrated with a marking ball prior to taking measurements.

### Statistical analysis

Descriptive statistics were calculated for all dogs enrolled in the study and are presented in Table 1, stratified by the presence of a TPLO reduction pin, and for the total study sample. Univariate and multivariate logistic regression models were estimated with the binary outcome for tibial tuberosity fracture. Multivariate models available in the Additional file 1: Figures S1–S3 included the following covariates: age (years), weight (kg), the presence of the reduction pin, and the difference in the angle pre- and post-op TPA angle. Results are presented in Table 2 as odds ratios, with 95% confidence intervals, and the associated p value. Statistical significance was achieved for p values less than alpha equal to 0.05. All statistical analyses were conducted using the software package R (version 3.2.3).

**Table 1:** Summary statistics of the study sample (presented figures are mean and standard deviations for continuous variables or frequency and percentage for categorical variables)

	With pin (n = 200)	Without pin (n = 200)	Overall sample (n = 400)
Age (years)	4.84 (2.63)	5.25 (2.49)	5.04 (2.57)
Sex (% female)	115 (57.5%)	101 (50.5%)	216 (54%)
Weight (kg)	31.29 (12.02)	32.46 (11.56)	31.88 (11.79)
Pre TPA angle	26.98 (5.18)	25.59 (3.78)	26.28 (4.58)
Post TPA angle	5.68 (3.98)	5.58 (3.28)	5.63 (3.64)
Post/pre angle difference	21.3 (5.21)	20.0 (4.11)	20.65 (4.73)
Tibial tuberosity width (cm)	0.94 (0.27)	0.94 (0.22)	0.94 (0.25)
Tibial width (cm)	4.14 (0.69)	4.28 (0.65)	4.21 (0.67)
Tibial tuberosity avulsion	1 (0.5%)	7 (3.5%)	8 (2%)

**Table 2:** Multivariate logistic regression (outcome: tibial tuberosity avulsion, where ‘1’ equals the event occurring)

Variable	Odds ratio	95% confidence interval		p value
Pin (ref = 0)	0.075	0.003	0.549	0.03832
Sex (ref = male)	0.927	0.208	3.653	0.916
Age (years)	1.050	0.779	1.391	0.73625
Weight (kg)	1.018	0.954	1.071	0.54327
Post/pre angle difference (degrees)	1.194	1.033	1.378	0.01218

### Results

A total of 400 dogs were included in the study that fit the criteria of 200 consecutive TPLO surgeries performed with each group were included in the study. Group 1 had 6 male intact, 76 male neutered, 3 female intact, 115 female spayed. Group 2 had 96 male neutered, 3 male intact, 101 female spayed, 0 female intact. The mean age during surgery was 4.8 years for group 1 and 5.2 years for group 2. The most common breeds were mixed breed (MBD) (37.5%), Lab (21%), and Pitbull (7.5%). Fractures occurred in English BD, Boxer, Lab (2), MBD, Beagle, and a Rottweiler. The median weight for group 1 was 29.65 kg (IQR: 24.58–37.02) and group 2 was 31.50 kg (IQR: 24.95–39.00). Additional details of groups 1 and 2 are presented in Table 1.

The 3.5 mm TPLO locking plate was the most common plate used by each group. Group 1 had 129 (64.5%) 3.5 mm plates, 31 (15.5%) 3.5 mm mini plates 20 (10.0%) 3.5 mm broad plates, and 20 (10.0%) 2.7 mm plates used. Group 2 had 141 (70.5%) 3.5 mm plates, 27 (13.5%) 3.5 mm mini plates, 17 (8.5%) 3.5 mm broad plates, and 15 (7.5%) 2.7 mm plates used. The TPLO reduction pin used for all 3.5 and 3.5 mm broad plates was a 5/64″ end threaded. The 0.062″ end threaded TPLO reduction pin was used for all 2.7 mm plates.

Based on the Student’s t-test there was a statistically significant difference in fractures observed in group 1 and group 2 with a significance level of p < 0.05. In univariate logistic regression analysis, only the covariate for the presence of the reduction pin was associated with a statistically significant reduction in the likelihood of tibial tuberosity avulsion [OR: 0.138 (95% CI 0.007–0.789)]. In the multivariate model, adjusting for potentially confounding covariates, the presence of the reduction pin was associated with an approximate 92% reduction in the likelihood of tibial tuberosity avulsion [OR: 0.075 (95% CI 0.003–0.549, p = 0.03732)]. Full results of the multivariate model are presented in Table 2.

In group 1, one fracture was observed in 200 consecutive TPLO surgeries (0.5%). The single fracture occurred in a 4-year-old Rottweiler with a pre-op TPA slope of 38° and post-op TPA slope of 9°. The absolute tibial tuberosity width was 0.76 cm and relative tibial tuberosity width was 0.16. The tibial tuberosity fracture was slightly displaced (less than 3 mm) with evidence of caudal rock back of the proximal segment.

In group 2 seven fractures were observed in 200 TPLO surgeries (3.5%). The mean age was 5 years, with a mean pre-op TPA of 30.2° and mean post-op TPA of 7.3°. The mean absolute tibial tuberosity width was 0.76 cm and relative tibial tuberosity width was 0.18.

### Discussion

To our knowledge this is the only study of TPLO procedures that evaluates the incidence of tibial tuberosity avulsion fractures in relation to the presence of an in situ TPLO reduction pin. Our data reveals a significant statistical difference between a TPLO performed with an in situ reduction pin and without the pin. No complications were reported due to the presence of the reduction pin in the time frame studied.

Tibial tuberosity fractures of 3.5% reported in group 2 (no pin) were in line with the previously reported tibial tuberosity fractures of 1–9% for TPLO’s performed without an in situ reduction pin [8]. In the last 15 years there have been six TPLO outcome studies each with over 90 patients that have been evaluated for tibial tuberosity fractures with TPLOs performed without the use of an in situ reduction pin. Combining all six studies together we have 1704 TPLO surgeries with an incidence of 3.6% (61) tibial tuberosity fractures [13–18]. In contrast, the only TPLO outcome paper to date that evaluated 1146 stifles and also had an in situ TPLO pin placement had a 0.4% (5) incidence of tibial tuberosity fractures [7]. This was similar to our incidence of 0.5% in group 1 (with pin).

The correct anatomic placement of the TPLO reduction pin with regard to the tibial crest and the effect on tibial tuberosity fractures has been previously reported. In this study, our only tibial tuberosity fracture with the in situ reduction pin occurred when the pin was improperly placed distal to Sharpey’s fibers. Placement of the pin distal to Sharpey’s fibers can weaken the tibial tuberosity and cause a stress riser [8]. Of the fractures in group 2, 71.4% (5/7) also had placement of the pin distal to the intended location.

Based on our results, we can conclude that an in situ TPLO reduction pin does decrease the incidence of tibial tuberosity fractures in our studied population. Although there are multiple factors associated with tibial tuberosity fractures, such as pin location and TPA, we believe the inclusion of a properly placed in situ TPLO reduction pin may be a simple technique that the surgeon can incorporate to decrease the incidence of tibial tuberosity fractures.

## Limitations

Limitations of this paper include the retrospective nature of the study and the potential selection bias. We attempted to limit selection bias by including all consecutive TPLO surgeries performed by one of the four board-certified surgeons and not limiting to a particular surgeon or particular size patient. It is possible that within our follow-up time tibial tuberosity fractures could have occurred and the owners elected care with their primary veterinarian or did not seek veterinary advice. In our experience, we have found that owners who have complications with a TPLO are more likely to follow-up with our hospital. Previous reports have found that tibial tuberosity fractures most likely occur before 5 weeks [11]. While it is very unlikely, tibial tuberosity fractures could have occurred after our 8-week minimum follow-up time.

## Additional file

**Additional file 1: Figure S1.** Boxplot showing age (years) by avulsion status. **Figure S2.** Boxplot of avulsion status by dog weight. **Figure S3.** Boxplot of avulsion status by the difference in pre- and post-op angle.

## Abbreviations

CCL: cranial cruciate ligament
TPLO: tibial plateau leveling osteotomy
TPA: tibial plateau angle
