# Supplementary figures and images for: Comparison of TPLO tibial tuberosity fractures with and without an in situ rotational pin
Source: BMC Res Notes. 2018 Jun 8;11:368. doi: 10.1186/s13104-018-3474-7 (PMC5994010; doi:10.1186/s13104-018-3474-7)

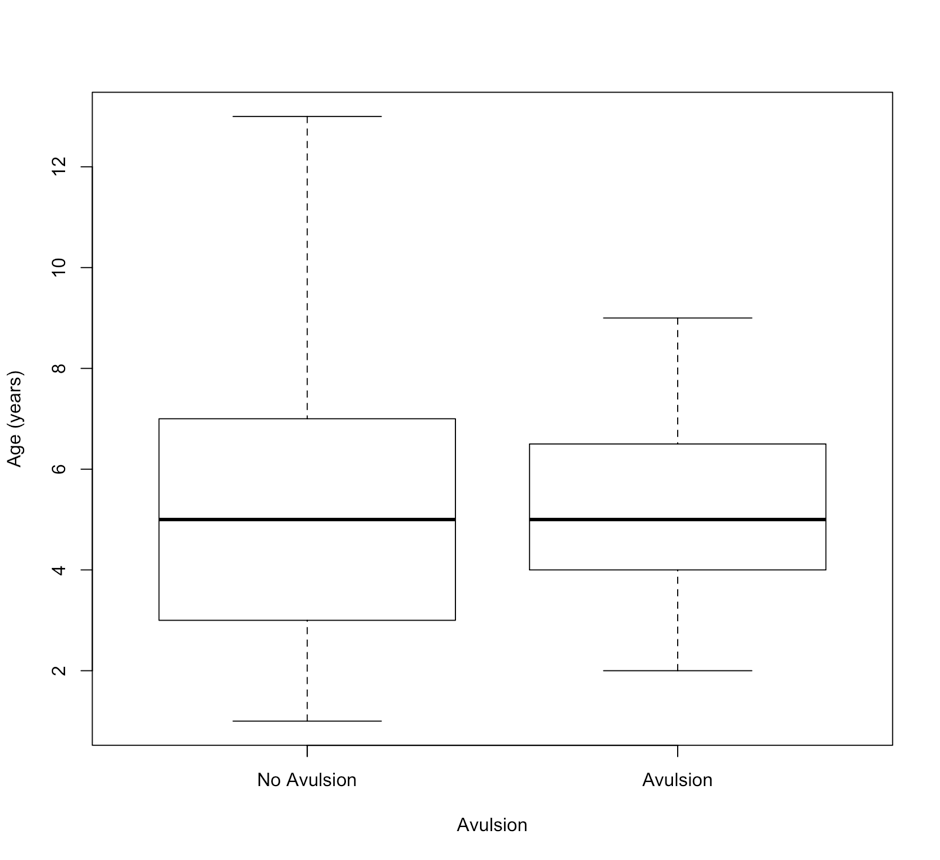


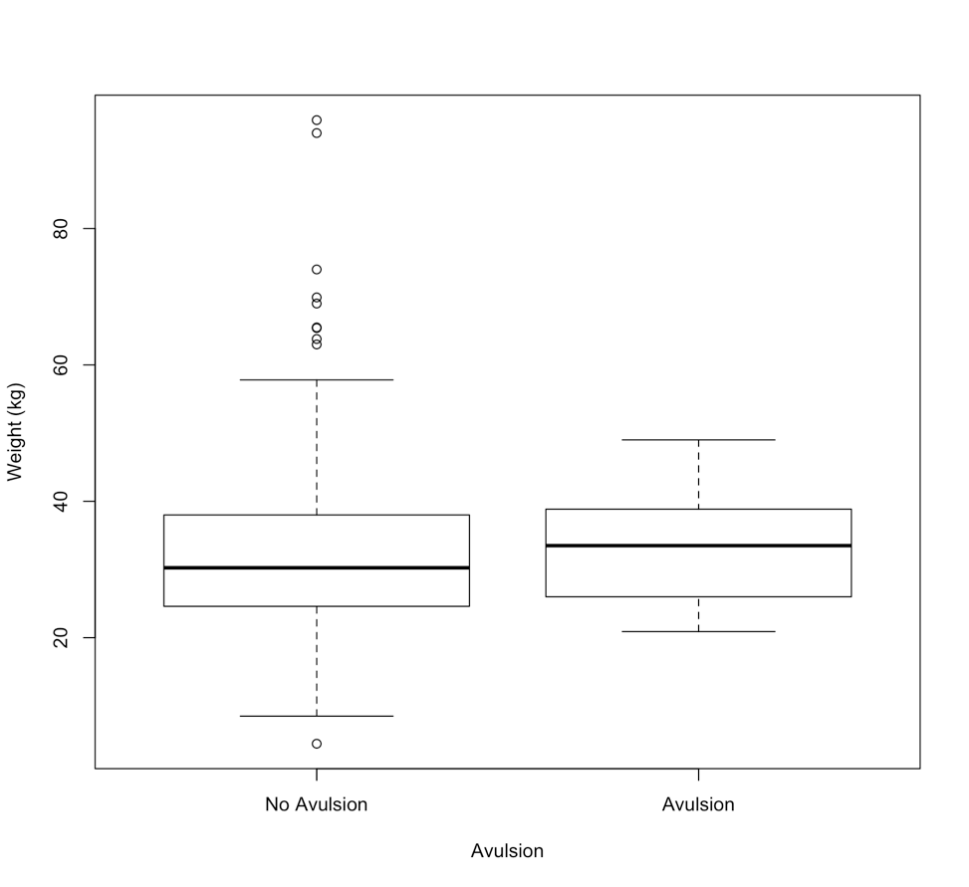


.


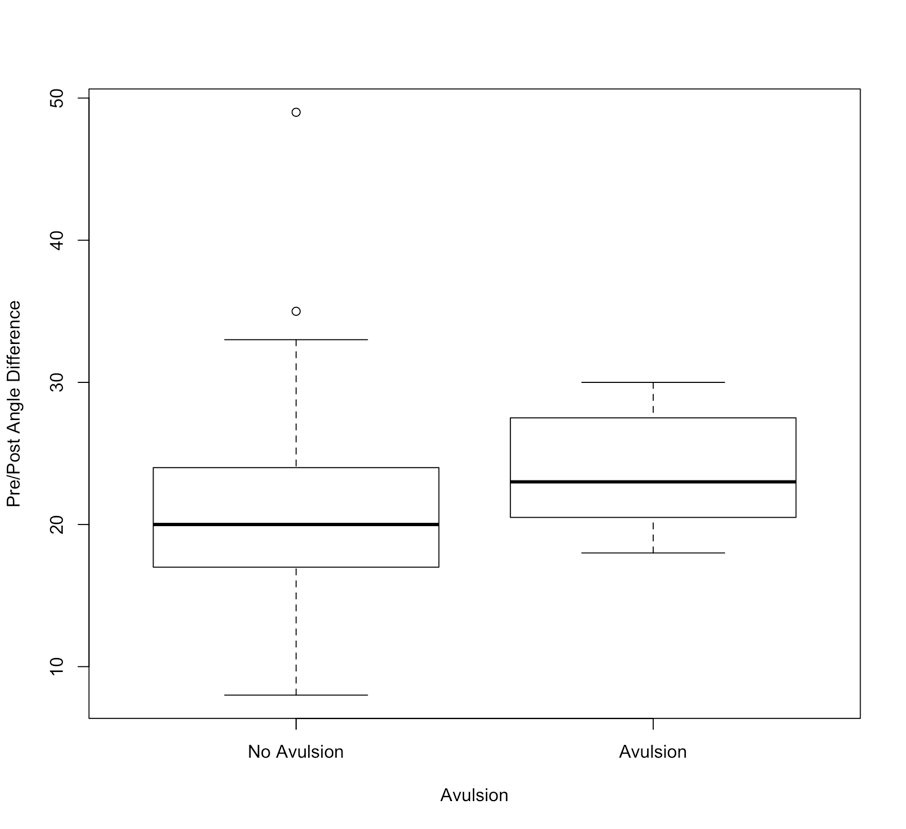

Supplement: Supplementary file 1 — Additional file 1: Figure S1. Boxplot showing age (years) by avulsion status. Figure S2. Boxplot of avulsion status by dog weight. Figure S3. Boxplot of avulsion status by the difference in pre- and post-op angle. [file 13104_2018_3474_MOESM1_ESM.docx]
